# MimiLook: A Phylogenetic Workflow for Detection of Gene Acquisition in Major Orthologous Groups of Megavirales

**DOI:** 10.3390/v9040072

**Published:** 2017-04-07

**Authors:** Sourabh Jain, Arup Panda, Philippe Colson, Didier Raoult, Pierre Pontarotti

**Affiliations:** 1 Aix-Marseille Université, Ecole Centrale de Marseille, I2M UMR 7373, CNRS équipe Evolution Biologique et Modélisation, 13284 Marseille, France; stararup@gmail.com; 2 Aix-Marseille Université, Unité de Recherche sur les Maladies Infectieuses et Tropicales Emergentes (URMITE), UM63 CNRS 7278 INSERM U1095IRD 198, Faculté de Médecine, 13284 Marseille, France; philippe.colson@univ-amu.fr (P.C.); didier.raoult@gmail.com (D.R.); 3 IHU Méditerranée Infection, Assistance Publique-Hôpitaux de Marseille, Centre Hospitalo-universitaire Timone, Pôle des Maladies Infectieuses et Tropicales Clinique et Biologique, Fédération de Bactériologie-Hygiène-Virologie, 13385 Marseille, France

**Keywords:** megavirales, giant viruses, HGT, gene acquisition, T-REX, workflow, orthologous groups

## Abstract

With the inclusion of new members, understanding about evolutionary mechanisms and processes by which members of the proposed order, Megavirales, have evolved has become a key area of interest. The central role of gene acquisition has been shown in previous studies. However, the major drawback in gene acquisition studies is the focus on few MV families or putative families with large variation in their genetic structure. Thus, here we have tried to develop a methodology by which we can detect horizontal gene transfers (HGTs), taking into consideration orthologous groups of distantly related Megavirale families. Here, we report an automated workflow MimiLook, prepared as a Perl command line program, that deduces orthologous groups (OGs) from ORFomes of Megavirales and constructs phylogenetic trees by performing alignment generation, alignment editing and protein-protein BLAST (BLASTP) searching across the National Center for Biotechnology Information (NCBI) non-redundant (nr) protein sequence database. Finally, this tool detects statistically validated events of gene acquisitions with the help of the T-REX algorithm by comparing individual gene tree with NCBI species tree. In between the steps, the workflow decides about handling paralogs, filtering outputs, identifying Megavirale specific OGs, detection of HGTs, along with retrieval of information about those OGs that are monophyletic with organisms from cellular domains of life. By implementing MimiLook, we noticed that nine percent of Megavirale gene families (i.e., OGs) have been acquired by HGT, 80% OGs were Megaviralespecific and eight percent were found to be sharing common ancestry with members of cellular domains (Eukaryote, Bacteria, Archaea, Phages or other viruses) and three percent were ambivalent. The results are briefly discussed to emphasize methodology. Also, MimiLook is relevant for detecting evolutionary scenarios in other targeted phyla with user defined modifications. It can be accessed at following link 10.6084/m9.figshare.4653622.

## 1. Introduction

Ever since the finding of the first giant amoeba virus Mimivirus [1,2], a thrust has been seen among researchers to understand the evolutionary mechanisms underlying their origin. With the discovery of new giant viruses in environmental samples over the past decade, representatives of new or putative new viral families namely Marseilleviruses, Pandoraviruses, Pithoviruses, Faustoviruses and Mollivirus [3,4,5,6,7] have been proposed to be included into a new order, Megavirales, together with viruses already classified in viral families *Ascoviridae*, *Asfarviridae*, *Iridoviridae*, *Phycodnaviridae*, and *Poxviridae*, and formerly known as nucleocytoplasmic large DNA viruses (NCLDVs). Members of the proposed order Megavirales have various genome sizes and infect a wide range of eukaryotic hosts [8,9,10]. This genome size variation (ranging from 100 kb to 2550 kb) has kept researchers questioning about their origin and evolution as these viruses challenge the established description of viruses and viral diversity. Certainly, they share a common ancestor, virion architectures and major functional features, as evident from the results of phylogenetic and phyletic analyses [10]. To explain this variation, several hypotheses have been put forward that postulate that either Megavirales were derived from a cellular ancestor after going through the process of genome degradation and adaptation [11,12], or, through expansion of viral genomes by acquisition of genes by the process of horizontal gene transfer (HGT) [13,14,15,16,17,18,19]. Regarding the latter case, the role of HGT in genome expansion cannot be discarded. However, genes transfer studies done so far have been more focused on understanding the evolution of a few Megavirale families (*Phycodnaviridae* and *Mimiviridae*) in particular, because of their genome variation and availability of several sequenced genomes. Apart from this, several studies have pointed out that the process of gene duplications [20,21], in addition to spreading of mobile genetic elements as introns or DNA transposons belonging to Polinton/virophage superfamily [22,23], can be evolutionary forces guiding the Megavirale genomic evolution.

Horizontal gene transfer involves a direct transfer of genetic material from one lineage to another. Main approaches used for detection of HGT in silico depend upon the analysis of sequence composition [24,25,26] or upon phylogenetic methods. Phylogenetic methods enable detection of gene transfers either (i) by reconstructing the gene tree and comparing it with the reference species tree, often via statistical analysis like bipartitions, quartet bipartitions, etc. [27,28]; or (ii) by examining gene history, through phyletic profiles [29]. Several algorithms like LatTrans [30], HorizStory [31], Efficient Evaluation of Edit Paths [32] and PhyloNet package including RIATA-HGT [33] and T-REX [34] are available to detect HGT events.

Members of the proposed order Megavirales have been shown over the past decade to be very common in our environment [35,36] and giant viruses were also detected in humans and suspected to be potential human pathogens [37,38,39]. Therefore, the need for understanding the major evolutionary forces guiding the evolution of Megavirale genomes and their gene history is growing, which makes it necessary to adopt some systematic searching for identifying the role of reticulate evolutionary events like HGT in evolution of distantly related Megavirale families. We herein describe an automated phylogenetic analysis workflow, MimiLook, that depends on the use of stringent filters to search systematically for probable instances of HGT in Megavirales. The workflow scripted in Perl connects existing programs to automate mining of conserved orthologous sequences among a designated number of Megavirale ORFomes, followed by a Basic Local Alignment Search Tool (BLAST) search to retrieve sequences of cellular domains of life that are homologous to these Megavirale clusters. The BLAST results are then subjected to alignment generation and phylogenetic tree (both gene tree and species tree) construction. Phylogenetic trees were then queried in T-REX [34] algorithm for detection of HGT instances. A reference Megavirale family tree was constructed from the orthologous groups’ information and various evolutionary scenarios are then mapped to this reference tree. Along with detection of HGT, the output of the workflow reveals various information about orthologous groups (OGs), such as which are Megavirale-specific and OGs which form monophyletic clades with other cellular domains of life. Some of the identified transfers are in accordance with previous analyses [40]. The results are discussed in brief to give more emphasis on development of strategy and methodology which is our main aim to present here.

## 2. A General Overview of Workflow

**Figure 1:**
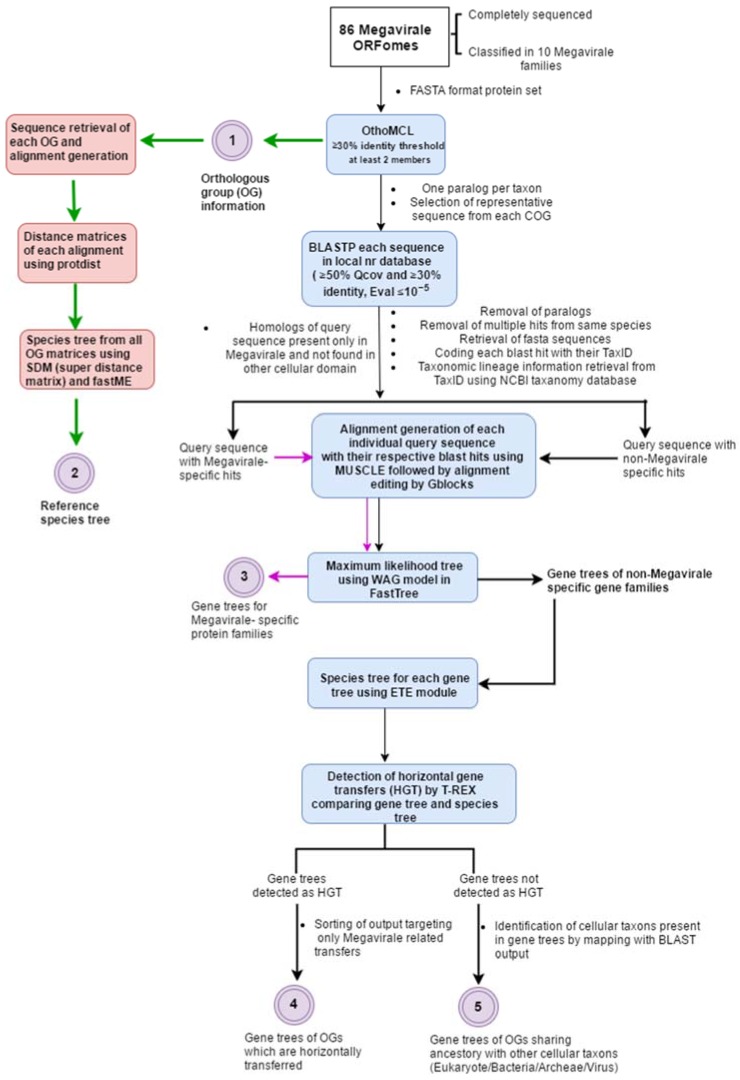
Depiction of workflow followed in MimiLook. Square colored boxes (in blue) indicate the calling of external executable algorithms in workflow. The bulleted text at each arrow and squared box without color indicates the steps which are written in Perl in this workflow. Colored circles with numbering are the outputs in each step of workflow (description of output labeled corresponding to them).

Our workflow inputs whole ORFomes in FASTA format (user-defined) and executes the following basic steps in sequential order: orthologous cluster identification, reference species tree construction, selection of representative sequence from cluster, searching for homologs of the representative sequence using protein-protein BLAST (BLASTP) in local database, filtering of the blast hits, retrieval of taxonomic lineage information, coding BLAST hits with taxonomy identifier (TaxID), alignment generation from filtered BLAST output, maximum likelihood (ML) tree generation from alignment, preparation of species tree for each gene tree, and finally, detection of HGT event (Figure 1). Some steps in the workflow involve automated curation and filtering of dataset for ease of use and understanding, which will be discussed in following paragraphs. The Megavirale family tree (reference tree) was constructed from resulting species tree and OGs are tagged on the tree manually, along with their evolutionary scenarios. The phylogenetic trees of each OG in Newick format are visualized using a Python script in Environment for Tree Exploration (ETE) phylogeny.

### 2.1. Complete ORFomes

The ORFomes for 86 completely sequenced members of 10 families (3 ascoviruses, 1 asfarvirus, 1 faustovirus, 16 iridoviruses, 3 marseilleviruses, 5 mimiviruses, 2 pandoraviruses, 18 phycodnaviruses, 1 pithovirus and 36 poxviruses) within the order Megavirales were downloaded from the National Center for Biotechnology Information (NCBI). The details regarding NCBI accession numbers and download link for these genomes are provided in Table S1.

### 2.2. Detection of Orthologous Groups

Complete ORFomes were queried in OrthoMCL [41] to retrieve orthologous protein groups with ≥30% identity and Expect (E) value of less than 0.00001 as thresholds. Only groups with members longer than 50 amino acids and at least two members were considered for further analysis. OrthoMCL is based upon all-against-all BLASTP algorithm [42] and Markov Cluster algorithm [43] that allows simultaneous classification of global relationships in a similarity space. OrthoMCL creates a similarity matrix from E-values and then clusters homologous proteins into related groups. The main parameter that influences the size of a cluster is the inflation parameter. A lower inflation parameter represents a more lenient clustering parameter (fewer clusters with more proteins) and a higher inflation parameter represents a more stringent clustering parameter (more clusters with fewer proteins). The inflation index of 1.5 was used to regulate cluster tightness (granularity), and the resulting clusters of orthologous groups were analyzed further (output 1).

Orthologous group information (output 1, Tables S1 and S2) was used to construct the reference Megavirale family tree using distance super-matrix approach. Amino acid sequences of each OG were retrieved from ORFome sets and they were further subjected to alignment generation using MUSCLE [44] followed by distance matrices generation by deploying ‘protdist’ program from Phylip package [45]. The computational efficiency and ease of use of distance-based tree reconstruction methods make them an attractive option for large data sets, where they can provide a snapshot of the underlying evolutionary relationships quickly and easily. A species tree was constructed from all gene matrices using the super distance matrix (SDM) approach—method weighted by alignment lengths [46]. A balanced minimum evolution tree was inferred from the resulting distance supermatrix by FastME (fast minimum evolution) [47], using NNI (nearest neighbor interchange), SPR (sub-tree pruning and grafting), and TBR (Tree Bifurcation and Reconnection) tree topology refinement (output 2). Further, Megavirale family tree topology was inferred from species tree and used as a reference tree for tagging various evolutionary scenarios (see Section 2.6). Many OGs will have multiple sequences for some proteins from same ORFomes, due to paralogy. In some analyses (e.g., gene family studies), paralogous sequences are informative, but, in this study, only one paralog per taxon is used to build the alignment for HGT detection in major orthologous groups. In these cases of two copies of orthologous genes in the same genome, we included the paralog with the best bit score in the corresponding cluster. After removal of paralogs from the OGs, we selected the representative sequence from each cluster—longest sequence in cluster was selected to increase the phylogenetic spread of homologs in non-redundant (nr) database—and carried out BLASTP search to detect homologs.

### 2.3. Search for Homologs and Curation of Blast Output

BLASTP [42] was used against local nr database (last accessed on 14 January 2016) to find potential homologs of each representative sequence (scoring matrix BLOSUM62, word size 3, low complexity region filtered out). An E-value cutoff equal to or less than 10^−5^ was set to filter out possible contaminants from blast output and hits below the threshold of 50% query size (coverage) plus 30% identity were further discarded. Since, we took one representative from each cluster, other sequences present in OG were automatically re-picked from nr database. A tabular output is generated with taxonomic lineage information of each significant hit (information such as TaxID, species name, etc., were retrieved). BLAST is currently the most common way of assessing homologous sequences, however, it can return identical hits (such as highly similar sequences from different strains of same species, allelic variations, paralogs, etc.) that do not add information to the analysis but instead make the final analysis more time-consuming.

Thus, to make BLAST tabular output more feasible and easy to use, we removed such sequences from the Blast output file. From hits of each queried sequence, paralogs from same species were removed by selecting the hit with the best bit score. Also, hits from different strains of same species were removed and one with the best bit score was selected. Amino acid sequences of significant hits were retrieved in FASTA format using blastdbcmd utility from nr database for each OG and taxonomic lineage information was extracted using corresponding TaxID. For keeping track of the names in this large dataset, we complimented the FASTA headers with their TaxIDs; thus, each OG had their unique homologous protein set in FASTA format coded with their TaxIDs. An annotation file in tabular format was prepared manually containing the TaxID of all Megavirale members retrieved from NCBI (both used and unused in dataset of our analysis; phages were excluded). Further, from homologous protein sets, those which contain Megavirale-specific hits and those containing non-specific Megavirale hits were separated, by comparing them with the annotation file. Both protein sets were used for further assessment in our workflow.

### 2.4. Alignment Generation, Alignment Editing and Tree Preparation

Both protein sets (containing amino acid sequences of optimal BLAST hits) were then queried in alignment program MUSCLE [44] to generate the alignment for each orthologous group, which is executed with the program’s default settings. After generation of alignment, Gblocks [48] was used for the removal of poorly aligned positions and highly divergent regions. Maximum likelihood (ML) trees from each such alignment were constructed using FastTree [49] using WAG [50] model of amino acid evolution. FastTree is optimized to work with large dataset and considered to be more accurate than many of other standard ML-based phylogenetic tree construction algorithms such as PhyML 3.0 or RAxML 7 [49,51]. At this stage of workflow, we were able to generate gene trees in Newick format of those OG which are specific (output 3) and non-specific to Megavirales. For further analysis, gene trees generated from non Megavirale-specific protein sets were queried in T-REX for detection of HGT event.

### 2.5. Detection of Horizontal Gene Transfer Event

Horizontal gene transfer detection by phylogenetic discordance method relies upon comparing gene tree with reference species tree. The trees generated by FastTree algorithm were used as gene trees for this analysis. Species trees were constructed for each gene tree separately. For this, we retrieved the TaxIDs of all the members of each alignment and fetched their common tree from NCBI taxonomy browser using those IDs. Each pair of species and gene tree was then subjected to T-REX [34] algorithm for inferring probable HGTs. T-REX is a suite of phylogenetic tools dedicated for several analyses including in-silico detection of HGT. Given a test and a reference tree, T-REX calculates their proximity by several distance-based measures and predicts minimum-cost scenario HGTs by progressive reconciliation of those trees. T-REX was optimized by taking into account the evolutionary events such as gene duplication, deletion, etc., and was attributed to be faster and more accurate than most of the other currently available tree discordance methods [34]. Further, this algorithm validates HGT by bootstrap and compilation of the consensus and interactive HGT scenarios. The output in text format contains name of the tree in which HGT is identified, donor branch, recipient branch and various statistical values like Robinson foulds distance, least square coefficient and bipartition dissimilarity. Since, our analysis only requires detecting HGTs either from Megavirales to cellular domains or from cellular domains to Megavirales; we modified the T-REX tabular output so that it contains only those types of detections. After curation, output 4 was generated containing the information of horizontally transferred gene families (OGs) with information of putative donor and receptor. Gene trees in which a HGT event was not detected were then further mapped with BLAST tabular output which contains taxonomic lineage information of each homologous hit, by which we are able to extract information about other cellular domains present in tree, thus generating output 5, which include gene trees of those OGs which have common origin with other cellular domains of life.

Gene trees which are generated at the end of this workflow (output 3, output 4, output 5) were in Newick format. So, in order to visualize the proper midpoint rooted and color coded phylogenetic trees, a python script was implemented in ETE toolkit [52]. For this purpose, another annotation file was prepared manually from taxonomic lineage information of Blast output. Here, for each taxon present in BLAST output, we prepared a tabular file containing columns with species name; cellular domain—Eukaryote, Bacteria, Archaea, Virus, Phages, Megavirales included in dataset, double-stranded DNA (dsDNA) (other Megavirales not included in dataset); group and lineage (highest possible taxonomic level).

### 2.6. Plotting OGs and Evolutionary Scenarios on Reference Megavirale Family Tree

Megavirale family tree topology was inferred from the reference species (output 2). Megavirale family specific OGs were plotted on this family tree considering presence/absence parameter for example, if a particular OG is present in at least one species of Megavirale family, then it is considered to be present in that family. Once the family specific OGs were attributed to their respective nodes, OGs shared between Megavirale families were than tagged on the tree. If a particular shared OG is present in multiple external nodes (family specific nodes), then it is judged to be present in common internal node (Figure S1). Further, evolutionary scenarios detected by our workflow (output 3, output 4 and output 5) were tagged on this tree too. The plotting was done manually using Microsoft Excel spreadsheet utility.

## 3. Implementation

MimiLook is a Perl (version 5.18.2) command line program developed on a 64-bit Linux architecture, currently running on Ubuntu 14.04.1 LTS. Before its commencement, programs like OrthoMCL [41], Muscle [44], protdist [45], SDM [46], fastME [47], Gblocks [48], FastTree [49], T-REX [34] were installed since the workflow requires calling these algorithms. More details of these programs are available in their respective installation documents. Local blast nr database was downloaded and installed. Also, the workflow requires ETE toolkit [52]. The input for MimiLook is a directory containing ORFomes to be analyzed in FASTA format and the final output is a directory containing gene trees (Newick format), blast tabular output, reference species tree (Newick format) and text file with information of HGT events. By using python script provided for plotting phylogenetic trees (10.6084/m9.figshare.4645273), png format trees were constructed (10.6084/m9.figshare.4645273). This workflow can be accessed from following link (10.6084/m9.figshare.4653622).

## 4. Results of MimiLook Analysis in the Proposed Order Megavirales

By using 86 ORFomes of Megavirales members classified in 10 families as input, this automated Perl command line program identified several gene families (i.e., OGs) which are specific to Megavirales and several other gene families sharing origin with cellular domains of life. The present dataset (containing nearly 29,000 proteins from all the ORFomes) took approximately 48 h on a 64-bit Linux machine to yield the output with minimal human intervention. The final results are stored in a directory containing various outputs enclosing information about the evolutionary scenarios of the OGs. We further evaluated the results according to the outputs generated in the various steps of this workflow.

**Figure 2:**
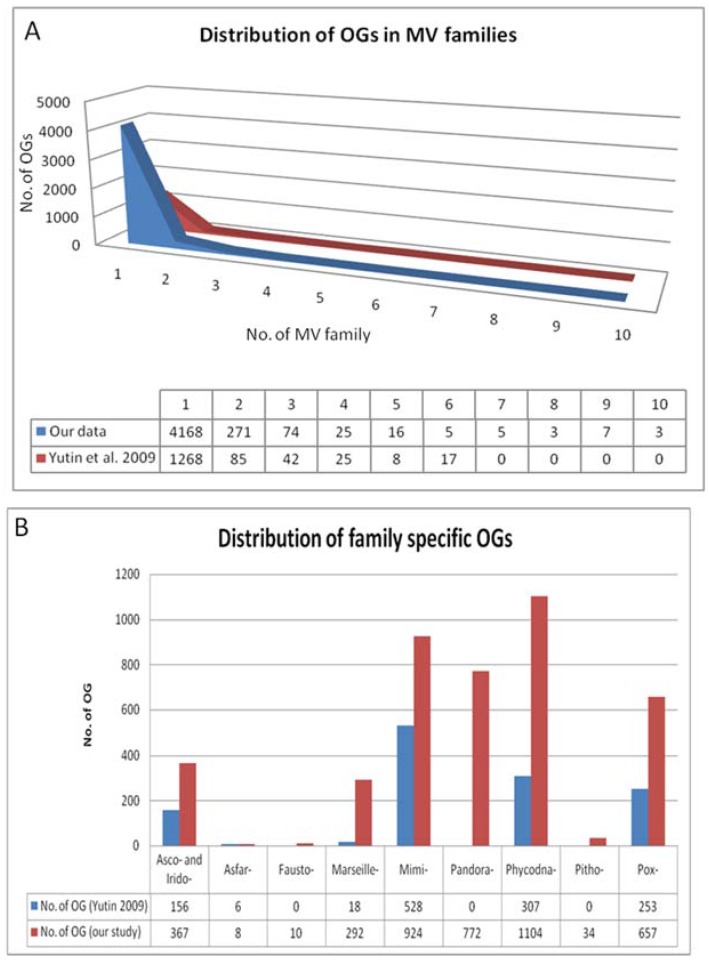
(**A**) Distribution of orthologous groups (OGs) in Megavirale families. OGs identified in our study (in blue) have been distributed in 10 families and compared with those identified in a previous study (in red). A large percentage of OGs are represented by only one Megavirale family (approximately 80% in both cases), whereas fewer OGs are shared between families. (**B**) Distribution of family specific OGs. *Phycodnaviridae* (Phycodna-) and *Mimiviridae* (Mimi-) contribute to the maximum number of family specific OGs in both studies. Our study (in red) identified 772 new OGs in pandoraviruses (Pandora-), 34 in pithovirus (Pitho-), 10 in faustoviruses (Fausto-), 657 in *Poxviridae* (Pox-) and 367 in *Ascoviridae* (Asco-) and *Iridoviridae* (Irido-) combined, as compared with the study done by Yutin et al. [9] (in blue).

Output 1: Of the 29,153 viral proteins encoded by the 86 Megavirale ORFomes, 21,256 proteins were clustered in 4577 OGs (Table S2). A total of 7898 proteins remained unclustered and not included in further analysis. The majority of OGs (4168) were found to be family specific (i.e., represented by species classified in one Megavirale family only), whereas, 409 OGs (approximately nine percent) were found to contain proteins from two or more Megavirale families (Figure 2A). Out of 4168 family specific OGs, 396 OGs were found to be represented by paralogs from same species i.e., they are present in only one species of one family (Table S3). An earlier study [9] was done with 45 NCLDV genomes from 7 families, whereas in this study 86 MV ORFomes from 10 families (three new putative families: faustoviruses, pandoraviruses and pithovirus) were included. Disparity is seen with the previously published NCVOG study mainly due to a larger dataset used in our analysis, but the pattern of results, such as large percentage of family specific OGs, large percentage of OGs shared by two species, more family specific OGs present in *Mimiviridae* and *Phycodnaviridae*, etc., are in accordance (Figure 2B).

**Figure 3:**
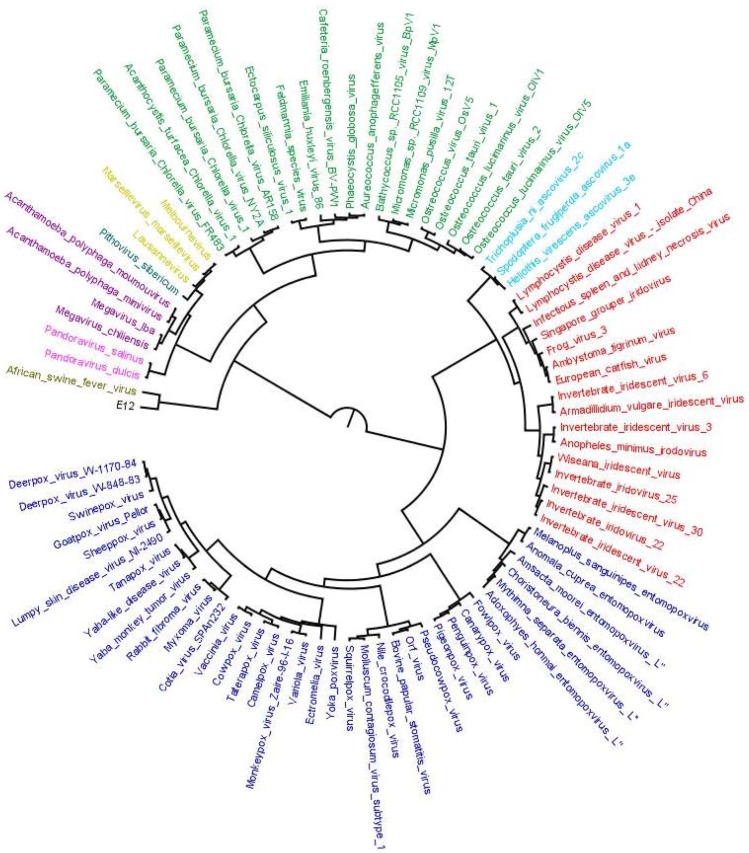
Distance-based species tree containing 86 representative members from 10 families of order Megavirales included in our study. Species tree is prepared by using SDM approach implemented on the orthoMCL output (output 1). The tree depicts two major clades: one clade with *Poxviridae* (blue), *Iridoviridae* (red) and *Ascoviridae* (aqua) and the other clade including *Phycodnaviridae* (green), marseillevirus (yellow), pithovirus (bluish green), *Mimiviridae* (Voilet), pandoravirus (pink) *Asfarviridae* (olive green) and faustovirus (black). Megavirale family tree inferred from this species tree is further used as guide tree for tagging evolutionary scenarios of OGs predicted by our workflow.

This output was further used to construct reference species tree based on SDM approach (output 2). The species tree prepared is shown in Figure 3. The tree depicts two major clades; one shows clade clusters *Poxviridae*, *Iridoviridae* and *Ascoviridae*, and the other, clade clusters faustoviruses, asfarviruses, marseilleviruses, mimiviruses, pithovirus and phycodnaviruses. This family tree has been used to tag various evolutionary scenarios predicted by our workflow. The next step in workflow identified homologs of each OG through BLASTP search.

**Figure 4:**
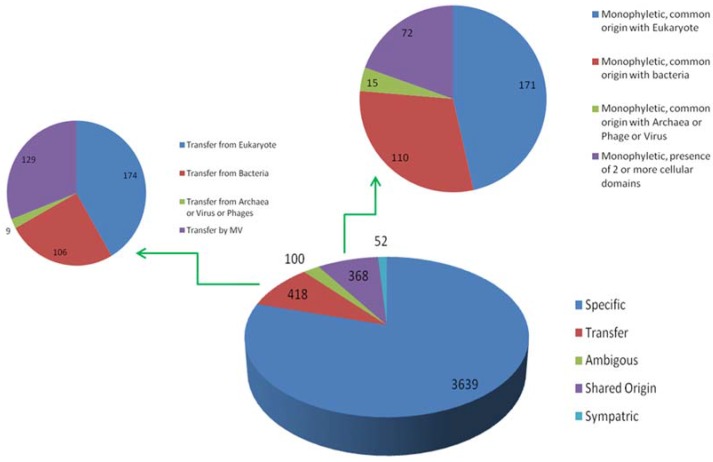
Evolutionary scenario to summarize results from output 3, output 4 and output 5. From total of 4577 OGs clustered, 3639 (80%) were found to be MV specific; 418 were involved in horizontal transfer (9%); 368 were sharing clades with homologs of other domains of life (8%), 100 (3%) were considered ambivalent and 52 had sympatric associations. Out of 418 transfers, 174 and 106 OGs were transferred to Megavirales by eukaryote and bacteria, respectively, while 129 were detected as probable transfer by Megavirales to other cellular domains. In addition, 171 and 110 OGs were found to be having monophyletic clade and commonly originating with Eukaryote or bacteria, respectively, whereas 15 were found to be having common origin with archaea, virus or phages. In 72 OGs, Megavirales are in monophyletic clade, but, there is presence of two or more cellular domains.

Output 3: This output includes those query sequences which have homologs present only in Megavirales. These hits were aligned and gene trees were prepared for each individual OG. We identified 3639 (approximately 80%) OGs (gene families) specific only to Megavirales, i.e., no homologs from organisms other than Megavirales are available in nr database by using our cut-off and stringent filters (Figure 4, Table S4).

Output 4: The remaining query sequences from BLAST output which were found to be non-Megavirale-specific, along with their respective homologous hits, were further analyzed for the detection of gene transfer event. Phylogenetic trees were generated after alignment and editing and subjected to detection of evolutionary events by T-REX algorithm. Out of 938 OGs, gene transfer was detected in 592 OGs (output 5). T-REX output (an example in Figure S2) contains information about putative donors and receptors along with statistical values of detected HGT, which we verified using human expertise by analyzing the graphical tree and identified 418 out of 592 OGs as probable cases of gene transfer (nine percent of total OGs) (Figure 4). Out of 418 OGs with detected HGTs, 174 (approximately four percent of the all OGs) were inferred to have transferred from eukaryotes, 106 (approximately two percent) gene families to have transferred from bacteria and nine gene families to have transferred from cellular domains other than eukaryotes or bacteria (archaea, and viruses, including phages). In some of the gene families, phylogenetic signal was not strong to identify the donor or to confidently predict the gene transfer event, so we put them in category ambivalent (100 OGs; approximately two percent). Fifty-two OGs were detected as cases of sympatric transfers (gene transfer by association of Megavirales with more than one cellular domain). Interestingly, 129 gene families (approximately three percent) were identified to be involved in gene transfers from Megavirales to other cellular domains (Table S4).

Output 5: Of the remaining 368 OGs which were not detected as transfer event, we mapped them with blast tabular output to identify the cellular domains with which they are sharing homology. Thus, we were able to detect 171 gene families which are monophyletic and share common origin with eukaryotes, 106 with bacteria, 15 with phages or other viruses or archaea and 72 monophyletic gene families sharing homologs in more than two cellular domains (Figure 4). 

**Figure 5:**
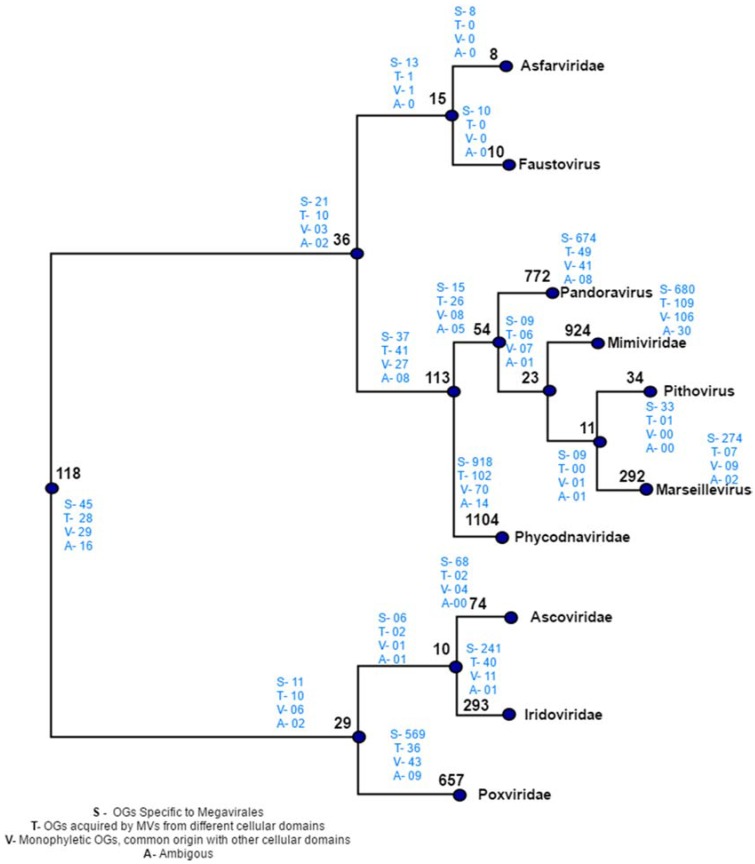
Distribution of orthologous groups and evolutionary scenarios on distance-based reference tree. Numbers written in black on each node depicts the total number of OGs present at that node. Numbers written in blue indicates the evolutionary scenarios predicted by our workflow.

Manual plotting of evolutionary scenarios and OGs distribution was done on the reference tree constructed by distance based method (Figure 5). The tree depicts two major clades: clade 1, clustering *Poxviridae*, *Ascoviridae* and *Iridoviridae*, and clade 2 clustering rest of seven Megavirale families. Out of 470 transfer events (418 HGT + 52 sympatric transfers), a total of 346 family specific OGs were detected, a majority of which are present in phycodnaviruses (102), mimiviruses (109) andpoxviruses (36), while fewer shared OGs (124) are involved in transfer event (Table S4). Similarly, large number of family specific OGs depicts Megavirale-specific evolutionary scenario, in comparison to shared OGS.

In order to check the specificity of our automated workflow, we compared our results with some of the already published gene transfers [40] and found few transfers in accordance with the previous study (Table 1), but, with a more specific description of gene acquisitions, i.e., putative donor/receptor of particular gene transfer (for protein IDs and corresponding orthologous group IDs (OG ID) see Table S2). These apparent differences may be attributed due to the large dataset (inclusion of three new Megavirale families) and robust methodology that we have applied to infer the evolutionary scenarios in this study.

## 5. Discussion and Future Perspective

Several approaches can be considered to detect incidence of HGT [26,27,28], however, the most convincing method to detect HGT uses phylogenetic inference [53]. Highly supported topological incongruence between a strongly supported gene tree and the known species tree can be penuriously explained by a reticulate evolutionary event like HGT [54]. Currently, few algorithms are available to detect HGT [29,30,31,32,33], but no workflow has been devised yet for searching systematically gene acquisition in large dataset of clustered orthologs. MimiLook described here is such an attempt to elucidate the evolutionary scenarios in user specific ORFomes, especially among MVs. It not only detects the instances of gene acquisition, but also allowed us to glance into other evolutionary patterns like phyla specificity and provided information about shared ancestry.

Phylogenetic analysis on orthologous clusters of Megavirales through our automated workflow MimiLook has detected a sizeable number of gene families which can be deduced as being acquired via HGT. Apart from this, we were also able to identify those gene families which are specific only of the order Megavirales and those gene families which share ancestry with cellular organisms (eukaryotes, bacteria, archaea), phages or other viruses. The main focus of this automated workflow is to detect reticulate evolutionary events like HGT by taking into consideration members from all the established or putative families of Megavirales. Interestingly, 129 gene families (OGs) have been found to be horizontally transferred by Megavirales to other organisms by our methodology, some of which are in accordance with what was described in previous studies [40,55,56,57] where acquisition of Megavirale genes by various eukaryotes has been shown. However, phylogenies of few gene families (approximately three percent) are ambivalent, because of a weak phylogenetic signal or complex phylogenetic patterns of transfers. So, in a further study, we could predict an evolutionary model of Megavirales by taking these pit falls in consideration, and the MimiLook has paved us a way for that by clustering distantly related Megavirale families and detecting evolutionary scenarios in orthologous gene families using stringent filters and statistical validation.

Gene families which are predicted as Megavirale-specific (80%) can be checked deeply and might be useful as genetic markers to identify new Megavirale species from environmental genomes and meta-genomes, considering the fact that a large fraction of those gene families are found family specific. By implementing this strategy, we can now have a closer look into this process.

Until recently, the impact of gene accretion in Megavirales was thought to be limited [13,14,15,16,17,18,19]. The reasons for this viewpoint included focus on a limited number of ORFomes of few families and the apparent reluctance of large-scale searches for HGT considering all available Megavirale ORFomes. This can be seen as a major shortcoming in interpreting the evolutionary forces guiding evolution of different distantly related families of Megavirales. So far, glancing into the output of MimiLook, the impact of gene accretion in Megavirales appears to be unlimited, which could have a profound effect on their evolution. Thus, our fully automated methodology which was developed here specifically for Megavirales, can give us more precise idea based on a more sensitive strategy about the evolutionary patterns among major families. Most importantly, using this methodology, we were able to infer putative donors and receptors in gene transfers and were able to obtain a more descriptive evolutionary pattern. Another important observation from the output is that some of the putative donors transferring genes are not known to be hosts of Megavirales, nor are they known to interact with them, thus suggesting the possibility of finding new ecological niches of them. Our results that the genomes of Megavirales members harbor many specific genes with no cellular homologs (approximately 80% Megavirale-specific OGs), as well as substantial numbers of genes inferred to have been transferred horizontally (approximately nine percent of OGs) from other cellular organisms, suggest that these viruses are not bags of unused genes taken from various cellular organisms, but, instead they might have acquired genes with a balanced process of genetic exchanges with their environmental interactions.

Although MimiLook was basically designed to detect evolutionary scenarios in Megavirales with an emphasis on reticulate events like HGT, ORFome sets can be selected according to the purpose of study. With little modification, we used this tool for detecting evolutionary scenarios in *Mycobacterium* species in our laboratory. MimiLook thus can be implemented for user specific sets of species to detect evolutionary events irrespective of a particular domain of life or viral group. Also, at each step, including clustering, alignment generation and editing, tree preparation and detection of HGT, algorithms and parameters can be modified according to the plan of the study.

## Figures and Tables

**Table 1:** Descriptive evolutionary scenarios detected by our automated workflow (in case of few published gene transfers).

ORF Name	Source of Transfer [40]	OG ID	Evolutionary Scenario Detected	Putative Donor/Receptor
**OtV1_33/OsV5_33**	Eukaryote	OG_02476	Transfer from eukaryote	*Magnoliophyta*
**OtV1_113/OsV5_131**	Eukaryote	OG_04266	Transfer from eukaryote	*Pyrenomonadales*
**OsV5_125**	Virus	OG_00455	Specific to Megavirales	-
**OsV5_218**	Eukaryote	OG_00469	Specific to Megavirales	-
**OtV2_3**	Bacteria	OG_00262	Shared origin with bacteria	-
**OtV2_29**	Bacteria	OG_04066	Transfer from bacteria	*Clostridiales*
**OtV2_30**	Bacteria	OG_00775	Transfer from bacteria	*Alphaproteobacteria*
**OtV2_40**	Ambiguous	OG_02248	Transfer from phages	Phages (*Myoviridae*)
**OtV2_78/OlV1_89**	Bacteria	OG_00556	Transfer from bacteria	*Helicobacter* (*Proteobacteria*)
**OtV2_158/OlV1_172**	Eukaryote	OG_02251	Shared origin with eukaryote	-
**OtV2_165**	Bacteria	OG_02131	Specific to Megavirales	MV specific
**OtV2_167**	Ambiguous	OG_02177	Shared origin with bacteria	-
**OtV2_179**	Virus	OG_04069	Shared origin with phages	-
**OtV2_193/OlV1_206**	Eukaryote	OG_02255	Specific to Megavirales	MV specific
**OtV2_201/OlV1_213**	Host	OG_02256	Transfer from eukaryote	*Saccharomycetes*
**OtV2_202/OlV1_214**	Ambiguous	OG_02257	Probable transfer	Probable transfer from *Rickettsiaceae* (bacteria)
**OtV2_222/OlV1_234**	Host	OG_01016	Transfer from eukaryote	*Mamiellales*
**OlV1_190**	Eukaryote	OG_02481	Sympatric transfer	Transfer from *Bacteria*; Gene with eukaryotes
**OlV1_236**	Host	OG_01062	Specific to Megavirales	MV-specific
**MpV1_8**	Host	OG_04280	Shared origin with eukaryote	-
**MpV1_9**	Host	OG_04281	Transfer from eukaryote	*Mamiellales*
**MpV1_201**	Bacteria	OG_00262	Transfer from bacteria	*Enterobacteriales* (independent acquisition)
**MpV1_203**	Virus	OG_00202	Transfer from Megavirales	Insectomime virus and Port-Miou virus
**Ny2A_137/AR158_126**	Ambiguous	OG_00853	Sympatric transfer	Transfer from *Chlorella variabilis*; Outside bacteria
**Ny2A_359**	Ambiguous	OG_00554	Ambiguous	Ambiguous (mix of bacteria and bacteriophages)
**Ny2A_543/AR158_487**	Ambiguous	OG_00554	Ambiguous	Ambiguous (mix of bacteria and bacteriophages)
**Ny2A_542/AR158_486**	Virus	OG_03082	Shared origin with phages	-
**Chiliensis_647**	Ambiguous	OG_01369	Transfer from Megavirales	*Brucellaceae* (bacteria)

ORF: Open reading frame.

## Supplementary Materials

The following are available online at www.mdpi.com/1999-4915/9/4/72/s1, Figure S1: Mapping OGs and evolutionary scenarios on reference tree, Figure S2: An example output of statistically validated HGT as predicted by T-REX and then verified by human expertise, Table S1: 86 Megavirale ORFomes used in this study, Table S2: Grouped Megavirale genes in cluster of orthologus groups, Table S3: Presence/absence matrix of orthologus groups in respective Megavirale species, Table S4: Predicted evolutonary scenarios in OGs of Megavirales.
