# Supplementary material for: MimiLook: A Phylogenetic Workflow for Detection of Gene Acquisition in Major Orthologous Groups of Megavirales
Source: Viruses. 2017 Apr 7;9(4):72. doi: 10.3390/v9040072 (PMC5408678; doi:10.3390/v9040072)
Supplement: Supplementary file 1 [file viruses-09-00072-s001.zip › viruses-184280_final-check_supplementary-1.pdf]

Supplementary Materials: MimiLook: A Phylogenetic Workflow for Detection of Gene Acquisitionin Major Orthologous Groups of Megavirales

Sourabh Jain, Arup Panda, Philippe Colson, Didier Raoult and Pierre Pontarotti

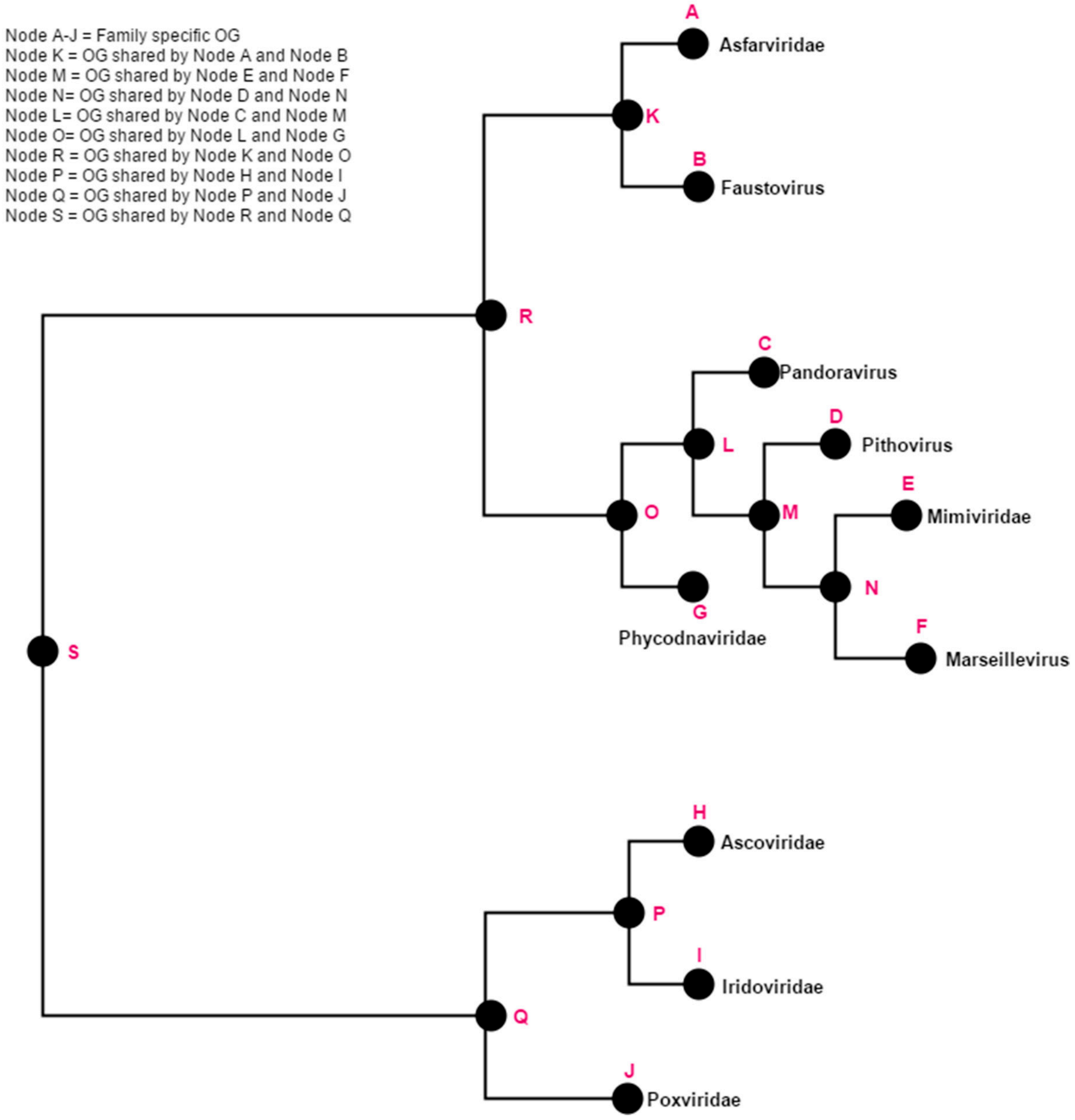

Figure S1. Mapping OGs and evolutionary scenarios on reference tree.Family specific nodes are labeled from A–J and internal nodes are labeled from K–S. At first, OGs which are present in family specific nodes (A–J) are plotted on reference tree, followed by the plotting of OGs present on internal nodes (K–S) (shown in figure label). After distribution of OGs, evolutionary scenarios are tagged using the OG present in each node.

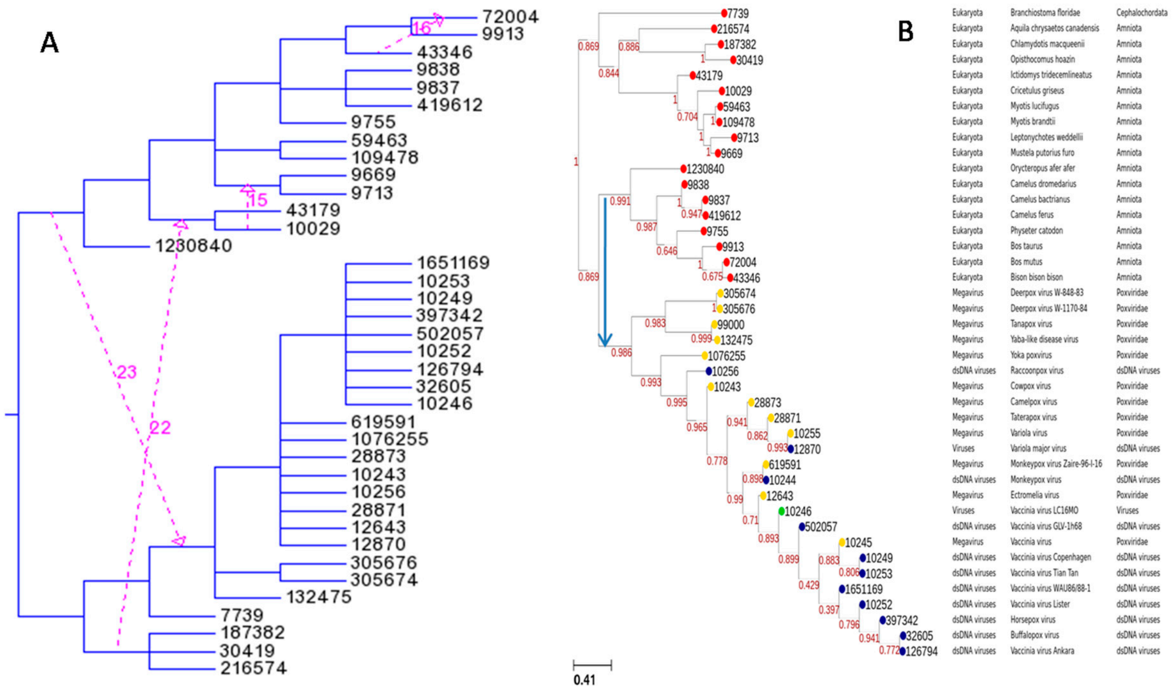

Figure S2. An example output of statistically validated HGT as predicted by T-REX and then verified by human expertise. In both trees, tip label is the TaxID of corresponding protein sequence. Scale below denotes substitutions per site. **(A)** HGTs detected by T-REX marked on species tree. Pink arrow denotes the direction of transfer. Total of four HGTs have been detected in this particular tree, but, as we are concerned about the transfers related to MVs only, thus, the transfer denoted as 23 was selected in our filtered output 5. **(B)** Gene tree prepared by blast output of single OG (midpoint rooted and ladderize descending). Here, now we can confirm the event of HGT from common ancestor of Eutheria (belonging to Amniota) to Megavirale (*Poxviridae*) (denoted by arrow), thus, this workflow paved as a way to detect putative donors and putative receptors in an HGT event.
